# Crystal structure of 4,4′-(disulfanediyl)dibutanoic acid–4,4′-bipyridine (1/1)

**DOI:** 10.1107/S1600536814018558

**Published:** 2014-08-30

**Authors:** Ana María Atria, Maria Teresa Garland, Ricardo Baggio

**Affiliations:** a Facultad de Ciencias Químicas y Farmacéuticas, Universidad de Chile, Casilla 233, Santiago, Chile; b Departamento de Física, Facultad de Ciencias Físicas y Matemáticas, Universidad de Chile, Santiago de Chile, Chile; c Departamento de Física, Centro Atómico Constituyentes, Comisión Nacional de Energía Atómica, Buenos Aires, Argentina

**Keywords:** crystal structure, hydrogen-bonding disposition, co-crystal

## Abstract

4,4′-(Disulfanediyl)dibutanoic acid (dtba) and 4,4′-bipyridine (4,4′-bpy) crystallize in an 1:1 ratio, leading to the title co-crystal with composition C_8_H_14_O_4_S_2_·C_10_H_8_N_2_. A distinctive feature of the crystal structure is the geometry of the dtba moiety, which appears to be stretched [with a 9.98 (1) Å span between outermost carbons] and acts as an hydrogen-bonding connector, forming linear chains along [-211] with the 4,4′-bpy moiety by way of O—H⋯N hydrogen bonds and C—H⋯O interactions. The influence of the mol­ecular shape on the hydrogen-bonding pattern is analysed by comparing the title compound and two other 4,4′-bpy co-crystals with closely related mol­ecules of similar formulation but different geometry, showing the way in which this correlates with the packing arrangement.

## Abstract 2

A distinctive feature of the crystal structure is the geometry of the dtba moiety, which appears to be stretched and acts as an hydrogen-bonding connector, forming linear chains along [-211] with the 4,4′-bpy moiety by way of O—H⋯N hydrogen bonds and C—H⋯O interactions. The influence of the mol­ecular shape on the hydrogen-bonding pattern is analysed by comparing the title compound and two other 4,4′-bpy co-crystals, showing the way in which this correlates with the packing arrangement.

## Chemical context   

The object of the present study, the 4,4′-(disulfanediyl)di­butan­oic acid mol­ecule C_8_H_12_O_4_S_2_ (dtba), consists of a ten-membered C(H_2_)_4_S_2_C(H_2_)_4_ chain setting apart the carb­oxy­lic acid groups at each end. This suggests that the mol­ecule may be a good candidate for a ‘spacer’ in the design of compounds with metal-organic framework (MOF) structures, provided that the mol­ecule connects the metal centres in an ‘extended’ fashion. However, the ‘solid-state shape’ of mol­ecules such as dtba is not directly discernible from first principles, as the chain includes many *sp* ^3^ carbon atoms, which may possibly lead to twisted linkages.
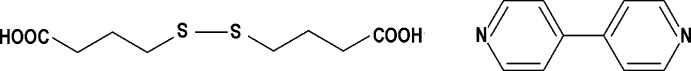


In addition, dtba is a rather uncommon ligand. The Cambridge Structure Database (Version 5.4, including June 2014 upgrades; Allen, 2002 ▶) does not at present include any entry whatsoever with the mol­ecule, either in its coordinating or free forms, for which any direct evidence of its shape is available. We have been trying for a while to coordinate the acid to some transition metals; however, so far we have been unsuccessful. During one of these numerous attempts, a co-crystal of dtba with 4,4′-bipyridine (4,4′-bpy), C_10_H_8_N_2_, was obtained instead. This serendipitous synthesis ended up being unique, since all subsequent attempts to obtain crystals with dtba ligand(s) in a more orthodox way have proved ineffective. We thus present herein the structural analysis of the 4,4′-bpy:dtba 1:1 co-crystal, C_8_H_14_O_4_S_2_·C_10_H_8_N_2_ (I), which to our knowledge is the first crystal structure to be reported surveying the dtba group.

## Structural commentary   

**Figure 1:**
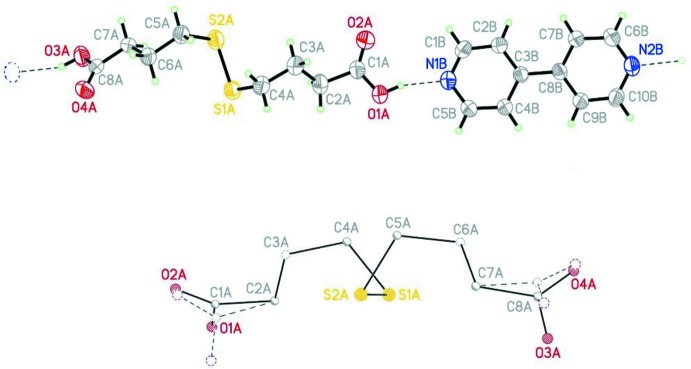
Top: the asymmetric unit of (I), showing the H—O⋯N linkages as dashed lines. Displacement ellipsoids are drawn at the 40% probability level. Bottom: the least-squares superposition of one dtba mol­ecule and its *C*2 image, showing the pseudo-symmetry in its central core.

Fig. 1 ▶ (top) presents an ellipsoid plot of the asymmetric unit of (I). The ‘topological’ (non-crystallographic) symmetry of the dtba mol­ecule with a twofold rotation axis located at the center of the S1—S2 bond is obvious from inspection, and it is somehow reflected in the bond-length sequence, presented in Table 1 ▶ (corresponding bonds are presented in the same line). In fact, the pseudo-symmetry goes a bit further: the group presents a non-crystallographic *C*_2*v*_ symmetry involving the mol­ecular core (C2–C7), which is reflected in the central torsion angles, *viz*. those involving S atoms (Table 1 ▶). Fig. 1 ▶ (bottom) shows the least-squares fit of this core and its *C*_2*v*_-related image, with deviations falling in the tight range 0.011–0.015 Å. The outermost parts of the mol­ecule (the carb­oxy­lic functions at the ends) deviate significantly from this trend, probably as a result of the strong O—H⋯N inter­actions with neighbouring 4,4′-bpy mol­ecules (see discussion below), a fact also reflected in the torsion angles involved (last two lines in Table 1 ▶). The double bonds in the –COOH groups are non-delocalized, with the C—O(H) bonds being distinctly longer than the C=O bonds (Table 1 ▶).

In spite of the unavoidable twisting due to the individual *sp* ^3^ carbon atoms in the chain, the mol­ecule can be considered to be stretched, with a C1⋯C8 span of 9.98 (1) Å and the terminal OH groups being almost anti-parallel to each other, subtending an angle of 175.5 (1)°. Thus, at least in the present structure, the mol­ecule can be considered as a potentially adequate spacer for MOF construction.

The 4,4′-bpy mol­ecule, in turn, is basically featureless, with slightly non-planar pyridine rings [maximum deviations from the least-squares planes: C2*B* 0.005 (3), N2*B*: 0.005 (3) Å], rotated to each other by 4.54 (13)°.

## Supra­molecular features   

**Figure 2:**
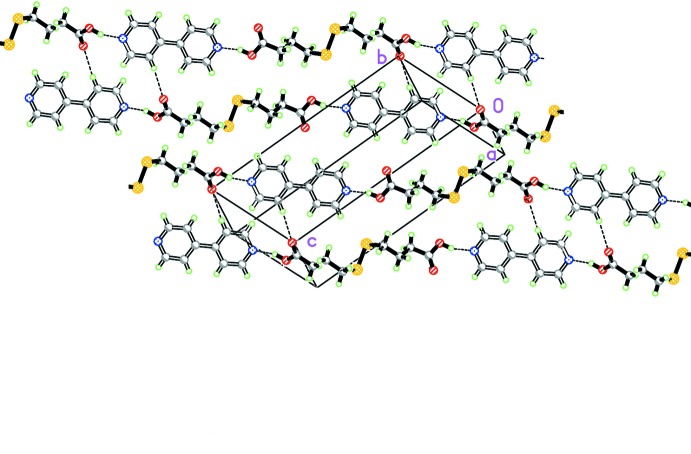
A packing view of (I), showing the slabs formed by neighbouring chains connected by C—H⋯O contacts (shown as dashed lines).

**Figure 3:**
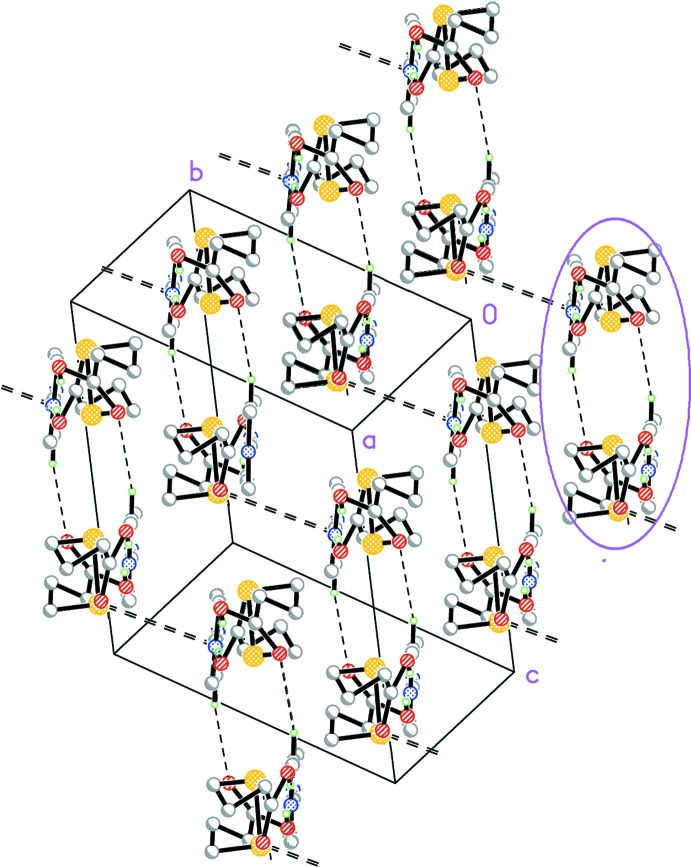
Packing view of (I) at right angles to the view in Fig. 2 ▶, showing the slabs in projection (one of them has been hightlighted). Single dashed lines denote the C—H⋯O bonds. The C—O⋯π contacts linking the slabs into a three-dimensional structure are shown as double dashed lines.

There are only two strong hydrogen-bond donors (the dtba carb­oxy­lic acid OH functions) and two hydrogen-bond acceptors (the pyridine N atoms of 4,4′-bpy) present, defining the supra­molecular organization (first two entries in Table 2 ▶) in the form of linear chains running along [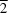
11] (Fig. 2 ▶) with graph-set descriptor 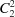
(22) (for graph-set nomenclature, see Bernstein *et al.*, 1995 ▶). Neighbouring chains, in turn, are connected into strips along [001] by a (notably weaker) C—H⋯O contact involving the pyridyl C9*A*—H9*A* group and one of the two non-protonated carboxyl­ato O atoms (third entry in Table 2 ▶, shown as nearly vertical broken lines in Fig. 2 ▶), giving rise to 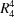
(16) centrosymmetric loops. The chains run parallel to each other, with no obvious second-order inter­actions linking them, either of the C—H⋯O, C—H⋯π or π–π types. There is, however, a different type of contact present, namely a C—O⋯π contact involving the non-protonated O atom [C1*A*—O2*A*⋯*Cg* ^i^ where *Cg*1 is the centroid of atoms N1*A*, C1*A*–C5*A*, symmetry code (i): 1 − *x*, 1 − *y*, 1 − *z*, with O2*A*⋯*Cg*1^i^ = 3.619 (3) Å; O2*A*⋯*Cg*1^i^, π: 165.25°], which helps in connecting the strips together into a three-dimensional supra­molecular structure (drawn in double dashed lines in Fig. 3 ▶). Thus, all potentially expected actors for the supra­molecular building (OH, O and N functionalities) end up fulfilling a relevant role in the overall organization.

## Database survey   

A brief search of the CSD confirmed that 4,4′-bpy:di­carb­oxy­lic acid adducts are rather frequent; among the most populated families, the one derived from alkanes/alkenes ranks on top. Many of these present ‘extended’ mol­ecular shapes, generating chain structures with similar O—H⋯N synthons as in (I). Among these, alkane-types are relevant to the present discussion as they are made up of *sp* ^3^ C atoms. There are cases with *n* = 5 (glutaric acid) and *n* = 6 (adipic acid; Pedireddi *et al.*, 1998 ▶), *n* = 7 (heptane-1,7-dioic acid; Braga *et al.*, 2008 ▶) and *n* = 10 (sebacic acid; Yu *et al.*, 2006 ▶).

**Figure 4:**
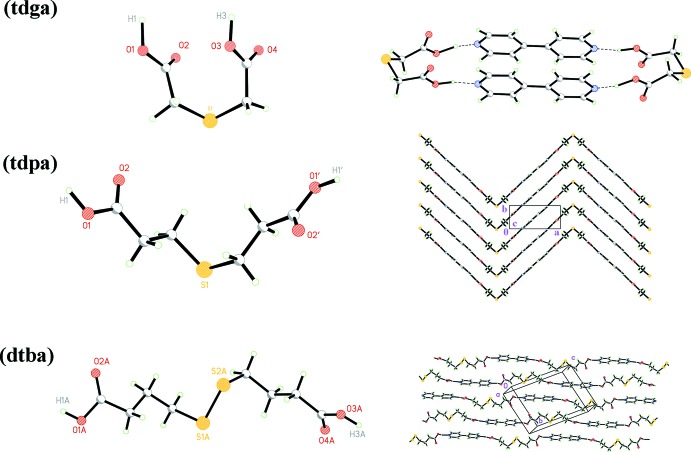
The three different mol­ecular shapes for tdga, tdpa and dtba, and the packing arrangements they give rise to, as described in the text.

However, examples of adducts with thio­dicarboxilyc acids are notably more rare and only two reported co-crystals of the sort can be found in the literature. These involve thio­dicarboxilyc acids closely related to dtba (see scheme below): thio­diglycolic acid (tdga) and thio­dipropionic acid (tdpa), *viz*. 4,4′-bpy:tdga and 4,4′-bpy:tdpa (Pedireddi *et al.*, 1998 ▶). Surprisingly, in these structures the linkers behave in a different way from dtba. Fig. 4 ▶ (left) shows the geometry of the three mol­ecules under discussion, while Fig. 4 ▶ (right) presents the packing arrangements they give rise to.
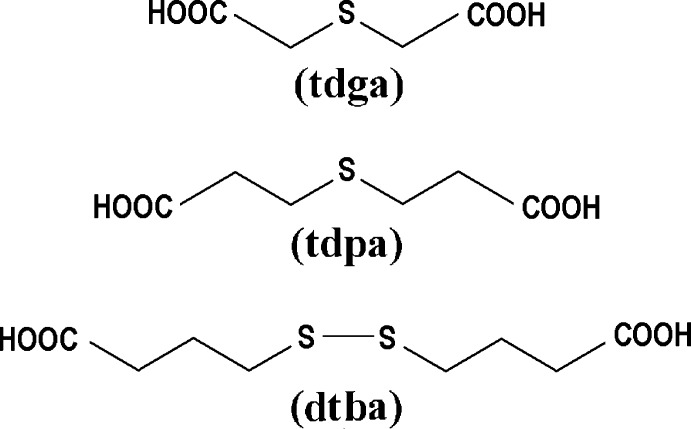


In the first case, (tdga co-crystal) the mol­ecule is shaped like a horseshoe, and the terminal OH functions end up being almost parallel, subtending an angle of 12.5 (1)° to each other. The 4,4′-bpy aggregation motifs with this particular geometry give rise to isolated closed dimers as shown in Fig. 4 ▶ (upper right).

The tdpa mol­ecule presents a shape somehow similar to, but noticeably more open than tdga, with H—O⋯O—H bonds almost at a right angle to each other [97.1 (1)°]. The resulting packing mimics this mol­ecular geometry, in a tight herring-bone pattern (Fig. 4 ▶, mid-right). Finally, and as already discussed, the present dtba co-crystal displays a fully stretched geometry [H—O⋯O—H: 175.5 (1)°] and the basic packing unit is a linear chain.

From this analysis it can be concluded (at least for this type of terminal dicarboxilic acids) that the relative angular disposition of the outermost OH groups are relevant in defining the expected general aspect of the packing. In this context, dtba could be considered a potentially useful spacer for MOF construction, and further work to obtain transition-metal complexes with this ligand is in progress.

## Synthesis and crystallization   

The reported 4,4′-bpy:dtba co-crystal was obtained seren­dip­it­ously from an unsuccessful synthesis of a holmium complex, prepared from an Ho_2_O_3_:dtba:4,4-bpy solution (in a 1:2:1 ratio), dissolved in a mixture of water (200 ml) and ethanol (20 ml). After a few days of slow evaporation at room temperature, colourless block-like crystals were obtained.

## Refinement   

Crystal data, data collection and structure refinement details are summarized in Table 3 ▶. All H atoms were originally found in a difference Fourier map, but treated differently in refinement: C—H H atoms were repositioned in their expected positions and thereafter allowed to ride with *U*_iso_(H) = 1.2*U*_eq_(host) (*d* = 0.93 Å for C—H_aromatic_ and *d* = 0.97 Å for C—H_methyl­ene_), while OH H atoms were refined with a restrained distance of 0.85 (1) Å.

## Supplementary Material

Crystal structure: contains datablock(s) I, global. DOI: 10.1107/S1600536814018558/wm5041sup1.cif

Structure factors: contains datablock(s) I. DOI: 10.1107/S1600536814018558/wm5041Isup2.hkl

CCDC reference: 1019479

Additional supporting information:  crystallographic information; 3D view; checkCIF report

## Figures and Tables

**Table 1:** Selected geometric parameters (Å, °)

C1*A*—C2*A*	1.513 (3)	C8*A*—C7*A*	1.497 (3)
C2*A*—C3*A*	1.487 (4)	C7*A*—C6*A*	1.507 (3)
C3*A*—C4*A*	1.522 (3)	C6*A*—C5*A*	1.522 (3)
C4*A*—S1*A*	1.811 (3)	S2*A*—C5*A*	1.805 (3)
C1*A*—O1*A*	1.309 (3)	O3*A*—C8*A*	1.325 (3)
C1*A*—O2*A*	1.198 (3)	O4*A*—C8*A*	1.197 (3)
S1*A*—S2*A*	2.0369 (14)		
			
C2*A*—C3*A*—C4*A*—S1*A*	−66.2 (3)	S2*A*—C5*A*—C6*A*—C7*A*	−67.6 (2)
C3*A*—C4*A*—S1*A*—S2*A*	−68.2 (2)	S1*A*—S2*A*—C5*A*—C6*A*	−67.67 (19)
O2*A*—C1*A*—C2*A*—C3*A*	−28.2 (4)	C6*A*—C7*A*—C8*A*—O4*A*	4.3 (4)
C1*A*—C2*A*—C3*A*—C4*A*	−165.0 (2)	C5*A*—C6*A*—C7*A*—C8*A*	178.3 (2)

**Table 2:** Hydrogen-bond geometry (Å, °)

*D*—H⋯*A*	*D*—H	H⋯*A*	*D*⋯*A*	*D*—H⋯*A*
O1*A*—H1*A*⋯N1*B*	0.85 (1)	1.83 (1)	2.661 (3)	163 (3)
O3*A*—H3*A*⋯N2*B* ^i^	0.86 (1)	1.80 (1)	2.637 (3)	162 (3)
C9*B*—H9*B*⋯O4*A* ^ii^	0.93	2.49	3.404 (3)	167

Symmetry codes: (i) 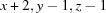; (ii) 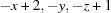.

**Table 3:** Experimental details

Crystal data	
Chemical formula	C_8_H_14_O_4_S_2_·C_10_H_8_N_2_
*M*_r_	394.49
Crystal system, space group	Triclinic, *P* 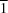
Temperature (K)	297
*a*, *b*, *c* (Å)	5.154 (3), 11.124 (7), 17.256 (11)
α, β, γ (°)	79.096 (10), 87.126 (10), 85.030 (12)
*V* (Å^3^)	967.3 (10)
*Z*	2
Radiation type	Mo *K*α
μ (mm^−1^)	0.30
Crystal size (mm)	0.23 × 0.14 × 0.11

Data collection	
Diffractometer	Bruker *SMART* CCD area detector
Absorption correction	Multi-scan (*SADABS*; Sheldrick, 2008*a* ▶)
*T*_min_, *T*_max_	0.94, 0.98
No. of measured, independent and observed [*I* > 2σ(*I*)] reflections	8306, 4185, 2376
*R*_int_	0.031
(sin θ/λ)_max_ (Å^−1^)	0.656

Refinement	
*R*[*F* ^2^ > 2σ(*F* ^2^)], *wR*(*F* ^2^), *S*	0.048, 0.131, 0.91
No. of reflections	4185
No. of parameters	243
No. of restraints	2
H-atom treatment	H atoms treated by a mixture of independent and constrained refinement
Δρ_max_, Δρ_min_ (e Å^−3^)	0.26, −0.16

Computer programs: *SMART* (Bruker, 2001 ▶), *SAINT* (Bruker, 2002 ▶), *SHELXS97*, *SHELXL97* and *SHELXTL* (Sheldrick, 2008*b* ▶) and *PLATON* (Spek, 2009 ▶).

## supplementary crystallographic information

### Crystal data

**Table:**

C_8_H_14_O_4_S_2_·C_10_H_8_N_2_	*Z* = 2
*M**_r_* = 394.49	*F*(000) = 416
Triclinic, *P*1	*D*_x_ = 1.354 Mg m^−^^3^
*a* = 5.154 (3) Å	Mo *K*α radiation, λ = 0.71073 Å
*b* = 11.124 (7) Å	Cell parameters from 1445 reflections
*c* = 17.256 (11) Å	θ = 2.4–21.1°
α = 79.096 (10)°	µ = 0.30 mm^−^^1^
β = 87.126 (10)°	*T* = 297 K
γ = 85.030 (12)°	Block, colourless
*V* = 967.3 (10) Å^3^	0.23 × 0.14 × 0.11 mm

### Data collection

**Table:**

Bruker SMART CCD area detector diffractometer	4185 independent reflections
Radiation source: fine-focus sealed tube	2376 reflections with *I* > 2σ(*I*)
Graphite monochromator	*R*_int_ = 0.031
CCD rotation images, thin slices scans	θ_max_ = 27.8°, θ_min_ = 1.2°
Absorption correction: multi-scan (*SADABS*; Sheldrick, 2008*a*)	*h* = −6→6
*T*_min_ = 0.94, *T*_max_ = 0.98	*k* = −14→14
8306 measured reflections	*l* = −22→22

### Refinement

**Table:**

Refinement on *F*^2^	2 restraints
Least-squares matrix: full	Hydrogen site location: mixed
*R*[*F*^2^ > 2σ(*F*^2^)] = 0.048	H atoms treated by a mixture of independent and constrained refinement
*wR*(*F*^2^) = 0.131	*w* = 1/[σ^2^(*F*_o_^2^) + (0.0623*P*)^2^] where *P* = (*F*_o_^2^ + 2*F*_c_^2^)/3
*S* = 0.91	(Δ/σ)_max_ < 0.001
4185 reflections	Δρ_max_ = 0.26 e Å^−^^3^
243 parameters	Δρ_min_ = −0.16 e Å^−^^3^

### Special details

**Table:**

Geometry. All e.s.d.'s (except the e.s.d. in the dihedral angle between two l.s. planes) are estimated using the full covariance matrix. The cell e.s.d.'s are taken into account individually in the estimation of e.s.d.'s in distances, angles and torsion angles; correlations between e.s.d.'s in cell parameters are only used when they are defined by crystal symmetry. An approximate (isotropic) treatment of cell e.s.d.'s is used for estimating e.s.d.'s involving l.s. planes.

### Fractional atomic coordinates and isotropic or equivalent isotropic displacement parameters (Å^2^)

**Table:**

	*x*	*y*	*z*	*U*_iso_*/*U*_eq_	
S1A	1.04515 (14)	−0.04804 (7)	0.29204 (4)	0.0744 (3)	
S2A	0.87636 (14)	0.02612 (6)	0.18771 (4)	0.0673 (2)	
O1A	0.7237 (4)	0.23802 (19)	0.51914 (11)	0.0781 (6)	
H1A	0.627 (5)	0.292 (2)	0.5388 (16)	0.105 (11)*	
O2A	0.4101 (4)	0.26892 (19)	0.43314 (12)	0.0884 (7)	
O3A	1.4882 (4)	−0.16886 (17)	−0.02741 (11)	0.0745 (6)	
H3A	1.596 (5)	−0.222 (2)	−0.0447 (18)	0.127 (13)*	
O4A	1.4099 (4)	−0.32979 (16)	0.06596 (10)	0.0775 (6)	
C1A	0.6119 (6)	0.2170 (2)	0.45711 (15)	0.0640 (7)	
C2A	0.7747 (6)	0.1205 (3)	0.42096 (16)	0.0794 (9)	
H2AA	0.9044	0.1604	0.3846	0.095*	
H2AB	0.8663	0.0643	0.4625	0.095*	
C3A	0.6193 (6)	0.0492 (3)	0.37810 (16)	0.0738 (8)	
H3AA	0.5658	0.1007	0.3287	0.089*	
H3AB	0.4630	0.0276	0.4095	0.089*	
C4A	0.7675 (6)	−0.0675 (2)	0.36052 (15)	0.0735 (8)	
H4AA	0.6464	−0.1145	0.3398	0.088*	
H4AB	0.8274	−0.1162	0.4100	0.088*	
C5A	0.7684 (5)	−0.1061 (2)	0.15574 (15)	0.0623 (7)	
H5AA	0.6608	−0.1495	0.1978	0.075*	
H5AB	0.6594	−0.0777	0.1106	0.075*	
C6A	0.9844 (5)	−0.1966 (2)	0.13303 (14)	0.0586 (6)	
H6AA	1.1029	−0.2205	0.1762	0.070*	
H6AB	0.9088	−0.2698	0.1247	0.070*	
C7A	1.1351 (5)	−0.1431 (2)	0.05930 (13)	0.0550 (6)	
H7AA	1.0154	−0.1211	0.0163	0.066*	
H7AB	1.2036	−0.0682	0.0675	0.066*	
C8A	1.3562 (5)	−0.2257 (2)	0.03466 (13)	0.0549 (6)	
N1B	0.4619 (4)	0.38299 (19)	0.60732 (12)	0.0634 (6)	
N2B	−0.2001 (4)	0.69792 (17)	0.89024 (12)	0.0619 (6)	
C1B	0.2496 (6)	0.4565 (3)	0.58974 (15)	0.0752 (8)	
H1B	0.1860	0.4652	0.5393	0.090*	
C2B	0.1163 (5)	0.5213 (2)	0.64262 (14)	0.0657 (7)	
H2B	−0.0307	0.5732	0.6270	0.079*	
C3B	0.2016 (4)	0.50899 (19)	0.71826 (12)	0.0482 (6)	
C4B	0.4247 (5)	0.4314 (2)	0.73679 (13)	0.0568 (6)	
H4B	0.4919	0.4201	0.7869	0.068*	
C5B	0.5455 (5)	0.3712 (2)	0.68026 (14)	0.0631 (7)	
H5B	0.6942	0.3194	0.6939	0.076*	
C6B	−0.2730 (5)	0.7174 (2)	0.81551 (15)	0.0655 (7)	
H6B	−0.4152	0.7733	0.8012	0.079*	
C7B	−0.1501 (5)	0.6598 (2)	0.75835 (14)	0.0578 (6)	
H7B	−0.2093	0.6767	0.7071	0.069*	
C8B	0.0639 (4)	0.57597 (19)	0.77791 (13)	0.0477 (6)	
C9B	0.1404 (5)	0.5567 (2)	0.85557 (13)	0.0577 (6)	
H9B	0.2827	0.5019	0.8715	0.069*	
C10B	0.0063 (5)	0.6186 (2)	0.90922 (14)	0.0633 (7)	
H10B	0.0622	0.6045	0.9608	0.076*	

### Atomic displacement parameters (Å^2^)

**Table:**

	*U*^11^	*U*^22^	*U*^33^	*U*^12^	*U*^13^	*U*^23^
S1A	0.0605 (5)	0.0877 (5)	0.0845 (5)	0.0148 (4)	−0.0181 (4)	−0.0456 (4)
S2A	0.0779 (5)	0.0572 (4)	0.0685 (4)	0.0008 (3)	0.0026 (4)	−0.0206 (3)
O1A	0.0807 (14)	0.0935 (14)	0.0625 (11)	0.0290 (11)	−0.0102 (10)	−0.0350 (11)
O2A	0.0810 (15)	0.1039 (15)	0.0861 (14)	0.0257 (12)	−0.0253 (12)	−0.0411 (12)
O3A	0.0912 (15)	0.0658 (11)	0.0640 (11)	0.0144 (10)	0.0107 (10)	−0.0185 (10)
O4A	0.0936 (15)	0.0618 (11)	0.0692 (12)	0.0252 (10)	−0.0073 (10)	−0.0048 (9)
C1A	0.0731 (19)	0.0640 (16)	0.0546 (15)	0.0076 (14)	0.0012 (14)	−0.0173 (13)
C2A	0.079 (2)	0.0869 (19)	0.0806 (19)	0.0143 (16)	−0.0110 (16)	−0.0441 (17)
C3A	0.075 (2)	0.0852 (19)	0.0631 (16)	0.0072 (15)	−0.0015 (14)	−0.0248 (15)
C4A	0.094 (2)	0.0654 (16)	0.0629 (16)	0.0057 (15)	−0.0097 (15)	−0.0197 (14)
C5A	0.0628 (17)	0.0658 (15)	0.0616 (15)	−0.0032 (13)	−0.0026 (13)	−0.0207 (13)
C6A	0.0675 (18)	0.0527 (13)	0.0574 (14)	−0.0010 (12)	−0.0042 (13)	−0.0154 (12)
C7A	0.0591 (16)	0.0546 (13)	0.0504 (13)	0.0096 (12)	−0.0096 (11)	−0.0114 (11)
C8A	0.0617 (16)	0.0589 (15)	0.0463 (13)	0.0086 (12)	−0.0143 (12)	−0.0183 (12)
N1B	0.0697 (15)	0.0664 (13)	0.0553 (12)	0.0062 (11)	0.0018 (11)	−0.0208 (11)
N2B	0.0729 (15)	0.0546 (12)	0.0586 (12)	−0.0003 (11)	0.0096 (11)	−0.0164 (10)
C1B	0.076 (2)	0.101 (2)	0.0514 (15)	0.0174 (17)	−0.0120 (14)	−0.0279 (15)
C2B	0.0632 (17)	0.0813 (18)	0.0520 (14)	0.0174 (14)	−0.0094 (12)	−0.0191 (13)
C3B	0.0533 (15)	0.0453 (12)	0.0456 (12)	−0.0007 (11)	−0.0001 (11)	−0.0089 (10)
C4B	0.0652 (17)	0.0558 (14)	0.0483 (13)	0.0105 (12)	−0.0059 (12)	−0.0125 (11)
C5B	0.0687 (18)	0.0602 (15)	0.0582 (15)	0.0149 (13)	−0.0047 (13)	−0.0136 (13)
C6B	0.0674 (18)	0.0630 (15)	0.0646 (16)	0.0115 (13)	−0.0004 (14)	−0.0158 (13)
C7B	0.0579 (16)	0.0642 (15)	0.0508 (13)	0.0078 (12)	−0.0047 (12)	−0.0140 (12)
C8B	0.0522 (14)	0.0415 (11)	0.0491 (12)	−0.0021 (10)	0.0021 (11)	−0.0092 (10)
C9B	0.0715 (18)	0.0498 (13)	0.0504 (13)	0.0080 (12)	−0.0070 (12)	−0.0103 (11)
C10B	0.086 (2)	0.0576 (14)	0.0460 (13)	0.0022 (14)	−0.0018 (13)	−0.0129 (12)

### Geometric parameters (Å, º)

**Table:**

C1A—C2A	1.513 (3)	C6A—H6AB	0.9700
C2A—C3A	1.487 (4)	C7A—H7AA	0.9700
C3A—C4A	1.522 (3)	C7A—H7AB	0.9700
C4A—S1A	1.811 (3)	N1B—C1B	1.320 (3)
C1A—O1A	1.309 (3)	N1B—C5B	1.330 (3)
C1A—O2A	1.198 (3)	N2B—C6B	1.334 (3)
S1A—S2A	2.0369 (14)	N2B—C10B	1.334 (3)
C8A—C7A	1.497 (3)	C1B—C2B	1.388 (3)
C7A—C6A	1.507 (3)	C1B—H1B	0.9300
C6A—C5A	1.522 (3)	C2B—C3B	1.377 (3)
S2A—C5A	1.805 (3)	C2B—H2B	0.9300
O3A—C8A	1.325 (3)	C3B—C4B	1.389 (3)
O4A—C8A	1.197 (3)	C3B—C8B	1.498 (3)
O1A—H1A	0.851 (10)	C4B—C5B	1.379 (3)
O3A—H3A	0.863 (10)	C4B—H4B	0.9300
C2A—H2AA	0.9700	C5B—H5B	0.9300
C2A—H2AB	0.9700	C6B—C7B	1.375 (3)
C3A—H3AA	0.9700	C6B—H6B	0.9300
C3A—H3AB	0.9700	C7B—C8B	1.393 (3)
C4A—H4AA	0.9700	C7B—H7B	0.9300
C4A—H4AB	0.9700	C8B—C9B	1.388 (3)
C5A—H5AA	0.9700	C9B—C10B	1.380 (3)
C5A—H5AB	0.9700	C9B—H9B	0.9300
C6A—H6AA	0.9700	C10B—H10B	0.9300
			
C4A—S1A—S2A	102.70 (10)	C6A—C7A—H7AA	108.5
C5A—S2A—S1A	102.95 (9)	C8A—C7A—H7AB	108.5
C1A—O1A—H1A	109 (2)	C6A—C7A—H7AB	108.5
C8A—O3A—H3A	108 (2)	H7AA—C7A—H7AB	107.5
O2A—C1A—O1A	123.6 (2)	O4A—C8A—O3A	123.4 (2)
O2A—C1A—C2A	126.0 (3)	O4A—C8A—C7A	125.4 (2)
O1A—C1A—C2A	110.4 (2)	O3A—C8A—C7A	111.2 (2)
C3A—C2A—C1A	113.4 (2)	C1B—N1B—C5B	116.8 (2)
C3A—C2A—H2AA	108.9	C6B—N2B—C10B	117.1 (2)
C1A—C2A—H2AA	108.9	N1B—C1B—C2B	123.4 (2)
C3A—C2A—H2AB	108.9	N1B—C1B—H1B	118.3
C1A—C2A—H2AB	108.9	C2B—C1B—H1B	118.3
H2AA—C2A—H2AB	107.7	C3B—C2B—C1B	120.0 (2)
C2A—C3A—C4A	113.2 (2)	C3B—C2B—H2B	120.0
C2A—C3A—H3AA	108.9	C1B—C2B—H2B	120.0
C4A—C3A—H3AA	108.9	C2B—C3B—C4B	116.6 (2)
C2A—C3A—H3AB	108.9	C2B—C3B—C8B	122.2 (2)
C4A—C3A—H3AB	108.9	C4B—C3B—C8B	121.2 (2)
H3AA—C3A—H3AB	107.8	C5B—C4B—C3B	119.4 (2)
C3A—C4A—S1A	116.6 (2)	C5B—C4B—H4B	120.3
C3A—C4A—H4AA	108.1	C3B—C4B—H4B	120.3
S1A—C4A—H4AA	108.1	N1B—C5B—C4B	123.8 (2)
C3A—C4A—H4AB	108.1	N1B—C5B—H5B	118.1
S1A—C4A—H4AB	108.1	C4B—C5B—H5B	118.1
H4AA—C4A—H4AB	107.3	N2B—C6B—C7B	123.8 (2)
C6A—C5A—S2A	115.37 (18)	N2B—C6B—H6B	118.1
C6A—C5A—H5AA	108.4	C7B—C6B—H6B	118.1
S2A—C5A—H5AA	108.4	C6B—C7B—C8B	119.4 (2)
C6A—C5A—H5AB	108.4	C6B—C7B—H7B	120.3
S2A—C5A—H5AB	108.4	C8B—C7B—H7B	120.3
H5AA—C5A—H5AB	107.5	C9B—C8B—C7B	116.6 (2)
C7A—C6A—C5A	112.23 (19)	C9B—C8B—C3B	121.9 (2)
C7A—C6A—H6AA	109.2	C7B—C8B—C3B	121.5 (2)
C5A—C6A—H6AA	109.2	C10B—C9B—C8B	120.3 (2)
C7A—C6A—H6AB	109.2	C10B—C9B—H9B	119.9
C5A—C6A—H6AB	109.2	C8B—C9B—H9B	119.9
H6AA—C6A—H6AB	107.9	N2B—C10B—C9B	122.8 (2)
C8A—C7A—C6A	115.2 (2)	N2B—C10B—H10B	118.6
C8A—C7A—H7AA	108.5	C9B—C10B—H10B	118.6
			
C2A—C3A—C4A—S1A	−66.2 (3)	C1B—N1B—C5B—C4B	−0.4 (4)
C3A—C4A—S1A—S2A	−68.2 (2)	C3B—C4B—C5B—N1B	0.3 (4)
O2A—C1A—C2A—C3A	−28.2 (4)	C10B—N2B—C6B—C7B	0.8 (4)
C1A—C2A—C3A—C4A	−165.0 (2)	N2B—C6B—C7B—C8B	−0.2 (4)
S2A—C5A—C6A—C7A	−67.6 (2)	C6B—C7B—C8B—C9B	−0.4 (4)
S1A—S2A—C5A—C6A	−67.67 (19)	C6B—C7B—C8B—C3B	178.8 (2)
C6A—C7A—C8A—O4A	4.3 (4)	C2B—C3B—C8B—C9B	175.5 (3)
C5A—C6A—C7A—C8A	178.3 (2)	C4B—C3B—C8B—C9B	−4.9 (4)
C6A—C7A—C8A—O3A	−175.9 (2)	C2B—C3B—C8B—C7B	−3.7 (4)
C5B—N1B—C1B—C2B	0.9 (5)	C4B—C3B—C8B—C7B	175.9 (2)
N1B—C1B—C2B—C3B	−1.2 (5)	C7B—C8B—C9B—C10B	0.3 (4)
C1B—C2B—C3B—C4B	1.0 (4)	C3B—C8B—C9B—C10B	−179.0 (2)
C1B—C2B—C3B—C8B	−179.5 (2)	C6B—N2B—C10B—C9B	−0.9 (4)
C2B—C3B—C4B—C5B	−0.5 (4)	C8B—C9B—C10B—N2B	0.4 (4)
C8B—C3B—C4B—C5B	179.9 (2)	O1A—C1A—C2A—C3A	152.4 (3)

### Hydrogen-bond geometry (Å, º)

**Table:**

*D*—H···*A*	*D*—H	H···*A*	*D*···*A*	*D*—H···*A*
O1*A*—H1*A*···N1*B*	0.85 (1)	1.83 (1)	2.661 (3)	163 (3)
O3*A*—H3*A*···N2*B*^i^	0.86 (1)	1.80 (1)	2.637 (3)	162 (3)
C9*B*—H9*B*···O4*A*^ii^	0.93	2.49	3.404 (3)	167

Symmetry codes: (i) *x*+2, *y*−1, *z*−1; (ii) −*x*+2, −*y*, −*z*+1.
